# Virulence of *Rigidoporus microporus* Isolates Causing White Root Rot Disease on Rubber Trees (*Hevea brasiliensis*) in Malaysia

**DOI:** 10.3390/plants10102123

**Published:** 2021-10-07

**Authors:** Wen Ze Go, Kit Ling Chin, Paik San H’ng, Mui Yun Wong, Chuah Abdullah Luqman, Arthy Surendran, Geok Hun Tan, Chuan Li Lee, Pui San Khoo, Wai Jern Kong

**Affiliations:** 1 Faculty of Forestry and Environment, Universiti Putra Malaysia, Serdang 43400, Selangor, Malaysia; wenze.go@gmail.com (W.Z.G.); waijern0525@gmail.com (W.J.K.); 2 Institute of Tropical Forestry and Forest Product, Universiti Putra Malaysia, Serdang 43400, Selangor, Malaysia; kitling.chin419@gmail.com (K.L.C.); chuah@upm.edu.my (C.A.L.); chuanli91kimi@gmail.com (C.L.L.); sansan_0928@hotmail.com (P.S.K.); 3 Faculty of Agriculture, Universiti Putra Malaysia, Serdang 43400, Selangor, Malaysia; geok_hun@upm.edu.my; 4 Institute of Plantation Studies, Universiti Putra Malaysia, Serdang 43400, Selangor, Malaysia; 5 School of Life Sciences, University of Warwick Wellesbourne, Warwick CV 359EF, UK; arthy.surendran@gmail.com

**Keywords:** molecular, pathogenicity, genetic phylogeny, virulence, white root rot pathogen

## Abstract

Latex production from *Hevea brasiliensis* rubber tree is the second most important commodity in Malaysia, but this industry is threatened by the white root rot disease (WRD) caused by *Rigidoporus microporus* that leads to considerable latex yield loss and tree death. This study aimed to characterize and compare the virulence of five *R. microporus* isolates obtained from infected rubber trees located at different states in Malaysia. These isolates were subjected to morphological and molecular characterization for species confirmation and pathogenicity test for the determination of virulence level. BLAST search showed that the ITS sequences of all the pathogen isolates were 99% identical to *R. microporus* isolate SEG (accession number: MG199553) from Malaysia. The pathogenicity test of *R. microporus* isolates conducted in a nursery with 24 seedlings per isolate showed that isolate RL21 from Sarawak has developed the most severe above- and below-ground symptoms of WRD on the rubber clone RRIM600 as host. Six months after being infected with *R. microporus*, RL21 was evaluated with the highest average of disease severity index of 80.52% for above- and below-ground symptoms, followed by RL22 (68.65%), RL20 (66.04%), RL26 (54.38%), and RL25 (43.13%). The in vitro growth condition tests showed that isolate RL21 of *R. microporus* has optimum growth at 25–30 °C, with the preference of weakly acidic to neutral environments (pH 6–7). This study revealed that different virulence levels are possessed among different *R. microporus* isolates even though they were isolated from the same host species under the same climate region. Taken together, field evaluation through visual observation and laboratory assays have led to screening of the most virulent isolate. Determination of the most virulent isolate in the present study is vital and shall be taken into consideration for the selection of suitable pathogen isolate in the development of more effective control measures in combating tenacious *R. microporus*.

## 1. Introduction

White root rot disease (WRD) is one of the most detrimental diseases in rubber plantations in Indonesia, India, Malaysia, Sri Lanka, Thailand, West and Central Africa [1]. This disease has been reported to cause about 3–15% of loss on production every year, particularly in small holder plantations [2]. According to the survey conducted by Sail et al. [3] in 2009, 43% of the small holder plantations in Malaysia were seriously infected with WRD. In Thailand, WRD is responsible for causing almost half of the yield losses in old rubber plantations, and killing rubber trees irrespective of age and health status [4]. Plant varieties within a species can differ in their ability to defend themselves. Numerous management methods have been introduced including clone screening, whereby rubber clones with higher resistance against WRD were recommended and bred for large scale *Hevea* plantation in Malaysia by the Rubber Research Institute of Malaysia (RRIM) [5].

Efforts in developing varieties with higher resistance to WRD are still proceeded by many other countries, in the hope to find a solution to suppress this disease. Despite the efforts of introducing higher resistance clones, *Hevea* plantations in the Southeast Asia are still facing significant economic loss due to the WRD, which reflects that the currently planted commercial clones are still susceptible to the WRD [6]. In most cases, infection rates of the pathogen on the host plant are highly influenced by the environmental conditions and the capacity of the plant to defend itself. However, under different circumstances, such as different degrees of isolates virulence in combination with the adverse environmental conditions which favor the pathogen, the interaction between the same plant variety and pathogen species may cause different outcomes.

*Rigidoporus microporus* is a well-known fungal pathogen, causing white root disease (WRD) on more than 100 different tree species. The greatest loss caused by this pathogen was recorded in rubber tree (*Hevea brasiliensis*) plantations [7]. It was first identified in 1904 as a pathogen of rubber tree in a botanical garden of Singapore [8]. Kaewchai et al. [9] conducted a pathogenicity test of WRD for 32 isolates of *R. microporus* obtained from two different provinces in Thailand. However, only 3 of the isolates obtained from Narathiwat Province were found to be highly virulent in causing WRD. Different isolates under the same pathogenic species may display different levels of virulence toward their host plant Lu et al. [10], which complicates the whole management strategies of WRD. Hence, information regarding the strains of white root rot pathogen present in certain locations, their virulence level, and genetic variations are of extreme importance to facilitate the development of efficient disease management strategies.

In the early 1980s, RRIM600 was the most common rubber tree clone planted in Malaysia and was recommended for wide scale planting in other rubber growing countries due to its wide adaptability, even in sub-optimal areas [11,12]. The clone RRIM600 is introduced as a high-yield cultivar but is also reported to be highly susceptible to white root rot disease [13]. Considering the significance of RRIM600 in the history of rubber tree breeding and its valuable phenotypic traits, the RRIM600 clone was used in this study. To date, information regarding the strains of white root rot pathogen present in Malaysia, their virulence level, and genetic variations are lacking. Therefore, isolates from symptomatic root tissues of rubber trees collected from different locations in Malaysia were characterized and their virulence against the rubber tree clone RRIM600 was determined in the nursery trial.

## 2. Materials and Methods

### 2.1. Source of White Root Rot Pathogen

Five pure fungal cultures isolated from rubber trees with WRD symptoms were obtained from the Laboratory of Crop Improvement and Protection Unit, Rubber Research Institute, Malaysia. Isolations were made from symptomatic root tissues of rubber trees collected from different locations in Malaysia (as shown in Table 1). The pathogen isolates were subcultured to a new petri plate of potato dextrose agar (PDA) (Difco^TM^, Becton, Dickinson and Company, Maryland, NY, USA), amended with streptomycin sulphate (300 mg/L) and chloramphenicol (180 mg/L) for bacterial suppression. The subcultures were then incubated at 28 ± 2 °C for further growth.

### 2.2. Morphological Characterization of White Root Rot Pathogen

Both macroscopic and microscopic were imaged using microscope (Eclipse E200, Nikon, Tokyo, Japan) with attached camera (DS-Ri1, Nikon, Tokyo, Japan). The macroscopic study was based on the colony features (color and texture) by referring to the publications by Farhana et al. [6], Kaewchai et al. [14], and Kaewchai and Soytong [1]. The microscopic study was performed by staining the mycelia lactophenol cotton blue (Merck, Germany) on a microscope slide using a sterile needle, according to the method used by Go et al. [15].

### 2.3. Molecular Identification of White Root Rot Pathogen

#### 2.3.1. DNA Extraction

The total genomic DNA of the white root rot pathogen was extracted according to the method by Lin et al. [16], with minor modifications. First, the actively grown fungal mass from the culture plate was carefully scraped with a fine sterilized spatula. About 100 mg of the fungal mycelia was weighed and used for the DNA extraction. The mycelia were then grinded using mortar and pestle, followed by the addition of 600 µL of ice-cold extraction buffer (100 mM Tris-HCl, pH 8.0; 40 mM EDTA, pH 8.0; 40 mM NaCl) at 28 °C. The mixture was transferred into a sterile 1.5 mL microcentrifuge tube. A 60 µL of sodium dodecyl sulphate and 12 µL of β-mercaptoethanol were added into the mixture followed by a brief vortex. The DNA mixture was then incubated in a water bath at 65 °C for 15 min. The incubated sample was mixed with 250 µL of phenol:chloroform:isoamyl alcohol (25:24:1) solution, followed by inverting the mixture and centrifuged at 11,000 rpm (the model of the machine, manufacturer, producing country) for 10 min at 28 °C. The supernatant was then transferred into a new 1.5 mL microcentrifuge tube. This step was repeated twice to completely remove the proteins/cell debris. An equal volume of ice-cold propanol was added into the supernatant and inverted to mix followed by incubation in ice for 10 min. After centrifuged at 4 °C, 11,000 rpm for 10 min, the supernatant was discarded and the DNA pellet was washed twice with 500 µL 70% ethanol, allowed to air dry in a laminar flow for 10 min and finally dissolved in 50 µL sterile distilled water. The extracted DNA was kept in −20 °C for further use. The extracted genomic DNA was quantified spectrophotometrically by measuring the absorbance at 260 nm using Multiskan GO microplate spectrophotometer (Thermo Fisher Scientific, Vantaa, Finland).

#### 2.3.2. PCR Amplification

The fungal ITS genes were then amplified using universal primers ITS1 (5′-TCCGTAGGTGAACCTGCGG-3′) and ITS4 (5′-TCCTCCGCTTATTGATATGC-3′) (White et al., 1990). A total reaction volume of 25 µL containing 12.5 µL of Taq Polymerase (1st BASE Biochemicals), 1.0 µL of template DNA, 1.0 µL for both forward and reverse primers with a concentration of 1.0 μM, and 9.5 µL of PCR grade distilled water was used for the amplification. The amplification reaction was performed using vapoprotect^®^ (Eppendorf, Germany) with the following specifications: initial denaturation for 2 min at 98 °C; 25 cycles of 15 s at 98 °C; 30 s at 60 °C and 30 s at 72 °C for annealing and extension, and final extension of the amplified DNA for 10 min at 72 °C.

The PCR products were visualized on 1% (*w/v*) agarose gel with FloroSafe DNA gel stain (1st BASE) as a substitute for ethidium bromide. Electrophoresis was run at 70 V for about 45 min to 1 h. The gel was visualized under UV Transilluminator. The image of the gel was captured with Gel Doc^TM^ XR System (BIO-RAD Laboratories, Hercules, California, USA).

#### 2.3.3. DNA Sequencing

The PCR products were then purified by Apical Scientific Pte Ltd. (Selangor, Malaysia) for direct DNA sequencing using BigDye^®^ Terminator v3.1 Cycle Sequencing Kit (Applied Biosystems). Sequences for each of the isolates were identified using Basic Local Alignment Search Tool (BLAST) searches (http://www.ncbi.nlm.nih.gov/BLAST, 19 May, 2018). All the new sequences of data of the isolated fungal pathogen were deposited in the GenBank nucleotide sequence databases.

#### 2.3.4. Phylogenetic Tree Analysis

The sequences of the white root rot pathogen isolates from this study, 16 related species of *Rigidoporus* (accession number: MG199553, MF574815, KX090082, KR076793, KJ654612, KJ654611, KJ559474, HQ400706, HQ400708, KJ559448, KJ559459, KJ559450, KJ559480, KJ831928, KJ559482, AY593868), and the outgroup *Oxyporus corticola* (accession number: KC176669) were used to construct the maximum-likelihood analysis. They were aligned manually using the ClustalW alignment tool in the Molecular Evolutionary Genetics Analysis (MEGA) 7 in the Alignment Explorer. Bootstrap confidence levels were adjusted to 1000 replicates. Evolutionary analyses were conducted in MEGA 7 [17].

#### 2.3.5. Pathogenicity Test

The pathogenicity of *R. microporus* isolates, namely RL20, RL21, RL22, RL25, and RL26, were evaluated on six-month-old rubber seedlings of the clone RRIM600 in the nursery of Faculty of Forestry, Universiti Putra Malaysia. The *R. microporus* inoculum was prepared using sterilized rubber wood block in the dimension of 6 cm × 6 cm × 6 cm. The fresh rubber wood blocks were oven-dried at 80 °C for 48 h and autoclaved for 30 min at 121 °C and 15 psi before the inoculation process. This sterilization step was repeated twice. Each of the rubber wood blocks was placed in high performance plastic (HPP) bags 17.8 cm × 25.4 cm, and 60 mL of molten malt extract agar (MEA) (Oxoid, UK) was added as a supplementary nutrient for the growth of *R. microporus*. About half the plate of *R. microporus* culture that has been cut into 1 cm^2^ plug was inoculated on the rubber wood block surface covered with solidified agar. The inoculated blocks were incubated under dark condition at 28 ± 2 °C for one month until they were fully colonized by *R. microporus* mycelium.

The rubber seedlings were then challenged with the five *R. microporus* isolates using sitting technique. Each seedling was inoculated with one of the five different *R. microporus isolates*. For each *R. microporus isolate* treatment, 24 rubber seedlings were inoculated using colonized rubber wood blocks. The rubber wood block inoculum was placed in contact with the roots of the seedling at the bottom of the polybags 25.4 cm × 30.5 cm, which contained 7 kg of sterilized growth medium consisting of soil, sand, and compost at a ratio of 4:4:1. Rubber seedlings grown in non-inoculated soil served as control.

#### 2.3.6. Disease Assessment

The disease development was observed at monthly interval for a period of six months. The Disease Severity Index (DSI) for each isolate were determined by applying the infection levels of above- and below-ground symptoms (as shown in Table 2) into Equation (1). This method was adapted from Wattanasilakorn et al. [18].

Disease severity index (DSI) % = (S_0_ × X_L1_) + (S_1_ × X_L2_) + (S_2_ × X_L3_) + …. (S_5_ × X_L5_) × 100 X_sum_ × S_H_ (1)

where:

S_0_, S_1_, S_2_…, S_5_: the disease severity scoring (0 to 4 for above-ground symptoms and 0 to 5 for below-ground symptoms)

X_L1,_ X_L2,_ X_L3_…, X_L5_: number of plants that are classified under the specific disease severity level

X_sum_: total of plants

S_H_: the highest scoring of disease severity classification for above-ground symptoms (4) or below-ground symptoms (5)

As shown in Table 2, the disease severity levels were categorized using the classification scheme with four disease severity levels for above-ground symptom and five disease severity levels for above-ground symptom. The root rot of inoculated seedlings was re-isolated to confirm the identity of the isolate. The most virulent isolate in the nursery trial was then selected for the cultural characteristic study.

### 2.4. Cultural Characterization of R. microporus Isolates

#### 2.4.1. Effects of pH on Mycelial Growth

The effect of pH on the growth of *R. microporus* isolates were tested using MEA medium with different pH levels. Agar plugs of 7 mm diameter containing mycelia of the pathogen were taken from actively growing margin of one-week-old culture and were inoculated on amended medium at different pH level (pH 3.5, 4.0, 4.5, 5.0, 5.5, 6.0, 6.5, and 7.0). The culture mediums were adjusted to the desired pH by adding 0.1 N NaOH or 0.1 N HCl. The pH value for each treatment was measured using a portable pH meter MW100 (Milwaukee Instruments, Rocky Mount, North Carolina, USA). The inoculated plates were kept in the incubator at 28 ± 2 °C and the diameter of the fungal colony was recorded daily. Five replicates were used for each treatment.

#### 2.4.2. Effects of Temperature on Mycelial Growth

The *R. microporus* isolates were inoculated on MEA plates and subjected to various temperatures (20, 25, 30, 35, and 40 °C) to evaluate the optimal growth temperature. The specific temperature condition was adjusted and controlled in an incubator and checked with a thermometer. Diameter of the fungal colony was recorded daily using an electronic calliper. Five replicates were used for each treatment.

#### 2.4.3. Effects of Light Regime on Mycelial Growth

The *R. microporus* isolates were exposed to different photoperiods of 24 h light, 24 h darkness, and 12 h light and 12 h darkness in a growth chamber maintained at 28 ± 2 °C with the selected medium, MEA, at pH 5.5. Inoculated Petri plates were kept in a growth chamber and light intensity was adjusted to the required level. The diameter of the fungal colony was recorded daily. Five replicates were used for each treatment.

### 2.5. Experimental Design and Statistical Analysis

The cultural characterization experiments were arranged with five replicates in a Completely Randomized Design (CRD). Quantitative data of mycelial growth was subjected to analysis of variance (ANOVA) with SPSS software (version 23). Tukey’s HSD test at 5% level of significance (*p* = 0.05) was used to compare the mean values of different treatments in the experiment.

## 3. Results

### 3.1. Morphological Characterization of White Root Rot Pathogen

**Figure 1:**
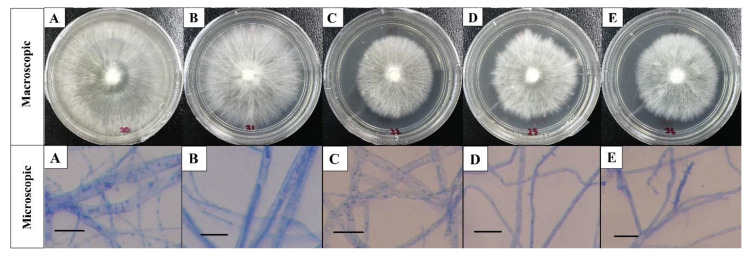
Macroscopic and microscopic view of white root rot pathogen isolates: (**A**) RL20, (**B**) RL21, (**C**) RL22, (**D**) RL25, (**E**) RL26 (Scale bar = 10 µm; magnification: ×40).

**Figure 2:**
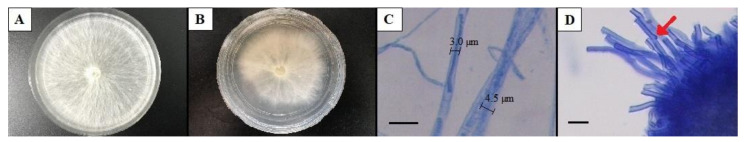
Characteristic of white root rot pathogen: (**A**) colony on PDA on the 8 DAI, (**B**) reverse side on PDA, (**C**) hypha and (**D**) generative hypha (arrow). (Scale bar = 10 µm; magnification: ×40).

There is no obvious difference for the macro- and microscopic observations among the white root rot pathogen isolates, as they showed similar types of colony characters and patterns (Figure 1). The mycelium of white root rot pathogen appeared to be white and flattened when cultured in PDA plate. PDA plates were fully covered within 8–10 days at 28 ± 2 °C (Figure 2A) with a milky color mycelium on the reverse side (Figure 2B). The width of hypha varied from 3.0 to 4.5 µm (Figure 2C). It was thick walled, hyaline, septate with no clamp connection, and hypha system was monomitic (generative hyphae) (Figure 2D).

### 3.2. Molecular Identification of White Root Rot Pathogen

The BLAST search showed that all ITS sequences of the white root rot pathogen isolates from RRIM were 99% identical to *R. microporus*, as all of them performed the highest level of similarity of 99% with isolate SEG from Malaysia (accession number: MG199553). The sequences were then submitted to GenBank and the accession numbers for each of the isolates are MN103601 for RL20, MN103602 for RL21, KM246744 for RL22, MN103603 for RL25, and MN103604 for RL26.

### 3.3. Phylogenetic Analysis

**Figure 3:**
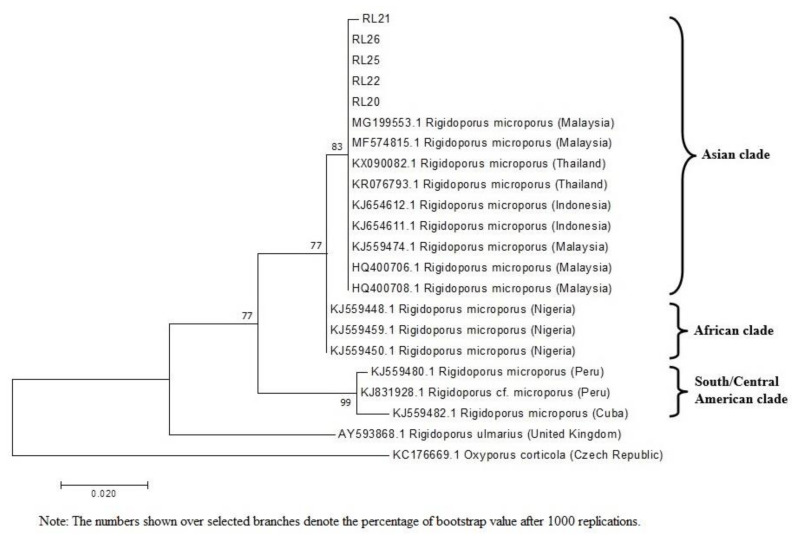
Evolutionary relationships based on the ITS region of the genomic rRNA gene of sixteen *Rigidoporus* isolates and one outgroup search derived from maximum likelihood analysis, showing the relationship between the five *R. microporus* isolates obtained with other closely related isolates and species.

The reference sequences available in the NCBI GenBank databases with a percentage of identity more than 90% were retrieved to compare with the ITS sequences of the white root rot pathogen isolates for the phylogenetic analysis. The tree that resulted from the phylogenetic analysis is presented in Figure 3. The ITS sequences of the studied isolates showed a close relationship with the isolates from Malaysia, Thailand, and Indonesia (Figure 3), with the highest level of similarity at 99% achieved by the isolate SEG (MG199553.1) which was identified as *R. microporus*. There were also three distinct clades represented in the phylogenetic tree among the selected *R. microporus* isolates. The Asian clade consisted of isolates from Malaysia, Thailand, and Indonesia. The African clade was represented by the isolates from Nigeria, while South or Central America clades were comprised of isolates from Cuba and Peru. The sequences of the RRIM isolates were divergent with the ITS sequence of *R. ulmarius* from the United Kingdom and outgroup *Oxyporus corticola* from Czech Republic. The constructed phylogenetic tree that supported the five white root rot pathogen isolates from RRIM had very high homology to known isolates from Malaysia, Thailand, and Indonesia, with a strong bootstrap value of 83 (Figure 3). White root rot pathogen isolates in this study were all identified and grouped together with other *R. microporus* isolates originating from Asia, which confirmed them as *R. microporus* with the match of their morphological and molecular characteristics.

### 3.4. Pathogenicity Test

**Figure 4:**
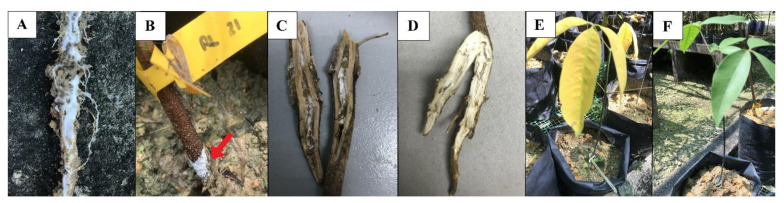
Signs and symptoms caused by *R. microporus* on rubber seedling: (**A**) full coverage of white rhizomorphs on the root surface at 1 MAI, (**B**) formation of white rhizomorphs on the collar region (arrow), (**C**) penetration of rhizomorphs to the root system, (**D**) healthy root from control seedling, (**E**) foliar symptom on rubber tree due to white root rot infection, and (**F**) healthy rubber tree with green leaves from control seedling.

*R. microporus* inoculated on the 6-month-old rubber seedlings clone RRIM600 showed the signs of WRD in the first month after inoculation (MAI), with rhizomorphs of fungus covering all areas of fibrous and lateral roots of the tree in the nursery trial (Figure 4A). The rhizomorphs are flattened mycelia strands of 1–2 mm thick that attached firmly to the surface of infected roots (Figure 4A). Through the observation, it was also shown that the white color rhizomorphs protruded around the collar region of the seedling at 1 MAI (Figure 4B). Furthermore, the white rhizomorphs were noticed to colonize the inner part of the root system, which was the most direct evidence in support of mycelia penetration into the root system of the rubber seedlings at 3 MAI (Figure 4C). The disease development was further confirmed by the root rotting process which turned the root structure black or dark in color (Figure 4C) in relation to the healthy root, which was bright in color at 3 MAI (Figure 4D). Besides that, the foliar symptom (yellowing) was noticeable on the seedlings infected by the white root rot pathogen at 3 MAI (Figure 4E), relative to the healthy rubber seedlings which had green leaves (Figure 4F). Seedlings showing foliar symptom died in less than two weeks. The above-mentioned symptoms and signs were visible on the RRIM600 seedlings infected by all the tested isolates with various degrees of infection. In order to confirm that the disease infection was caused by *R. microporus*, the pathogen was then re-isolated onto MEA plates from the rotted roots of the artificially inoculated seedlings. The cultural characteristics of the re-isolated fungus were similar to the original isolate.

### 3.5. Virulence of R. microporus Isolates

Differences in virulence among the selected isolates were determined based on the level in leaf yellowing, root necrosis, and a mortality rate of intervals up six months (Table 3 and Table 4). At 1 MAI, all the isolates developed rhizomorphs on the surface of the tap roots of all inoculated seedlings, while the foliar symptom slowly developed at 3 MAI on the cultivar RRIM600. The disease severity scoring based on the foliar discoloration and percentage of root infection developed at 6 MAI has demonstrated that *R. microporus* isolate RL21 managed to cause the disease development to the highest severe level (DSI = 80.52%) (Table 3) in the rubber seedlings, with its mortality rate after 6 MAI recorded at 83.33% (Table 4). These results showed that the RL21 isolate was the most destructive to the host, followed by RL22 (68.65%), RL20 (66.04%), RL26 (54.38%), and RL25 (43.13%) in terms of DSI of above- and below-ground symptoms. RL25 and RL 26 isolates were less virulent (Table 3) towards the RRIM600 rubber host. The foliar symptom was supported by the disease severity index of the root infection (Table 3), in which necrosis or rotted root conditions were used as the indicator (Figure 4C). The mortality rate on the rubber clone RRIM600 has revealed that most of the isolates caused serious damage to the host at 3 to 4 MAI. According to the pathogenicity test, the lowest mortality rate of seedling was recorded on isolate RL25, with half of the seedlings able to survive until the end of the experiment (Table 4). Isolate RL21 denoted the highest mortality rate on the selected host, which was recorded at 16.67%. There was no visible formation of fruiting bodies on the collar region of the tested seedlings, even on the 6 MAI. The results obtained from this pathogenicity test, as based on the disease severity index and the survival rate, suggested that the isolate RL21 of *R. microporus* was the most virulent in causing WRD infection on the RRIM600 rubber seedlings.

### 3.6. Cultural Characterization of R. microporus Most Virulent Isolate

#### 3.6.1. Effects of pH on Mycelial Growth

The growth of *R. microporus* isolates was tested at pH values, ranging from 3.5 to 7.0 on MEA medium. The response of the fungus towards different pH levels is shown in Table 5. Different pH value had a significant effect on the radial growth of all the isolates. Of all the eight pH levels, cultivation medium with pH 6.5 had the most significant effect on the colony radial growth, with the largest colony size of 55.57 mm recorded for isolate RL21 at 3 DAI. Radial growth of the colonies for all isolates were slower with lower pH. Radial growths of the colonies were more rapid in medium condition that producing moderate mycelial density when compared with those producing poor or abundant mycelial density. Medium with lower pH promoted thicker mycelial density, but radial growth was slower, causing small colony diameters, whereas medium with higher pH caused lower mycelial density but higher radial growth. A detrimental effect on the mycelial growth rate reduction was observed at pH 3.5. Among all the isolates, higher radial growths were observed in RL21 and RL22 regardless of the pH levels.

#### 3.6.2. Effects of Temperature on Mycelial Growth

The influence of various temperatures on the growth of *R. microporus* isolates were depicted in Table 6. The colony growths for all isolates were significantly the highest, at 30 °C. Radial growths were significantly weaker when incubation temperature was below 25 °C and above 35 °C. No growth was observed in the culture plates kept at 40 °C, even after six days of incubation period. This indicated that high temperature of 40 °C suppressed the growth of *R. microporus* isolates.

#### 3.6.3. Effects of Light Regime on Mycelial Growth

Average colony diameters of all five isolates of *R. microporus* after third days of incubation under different photoperiods are given in Table 7. There was no significant difference in the radial growth for all isolates, except RL22, when exposed to different photoperiods. This reflected that isolates RL20, RL21, RL25, and RL26 grew equally regardless of the effect of photoperiods (alternate in light-dark, 24 h in darkness, and 24 h in light exposure). The inhibitory effect of light on the growth of isolate RL22 was observed in this study. When compared to light incubation, dark incubation induced more rapid radial growths for isolate RL22. Lower mycelial density, as well as slower radial growth, were observed in isolate RL22 when 24 light incubation was applied.

## 4. Discussion

Numerous reports have been published about the occurrence of white root rot disease on rubber trees from countries like Thailand, Sri Lanka, Indonesia, and Nigeria. However, only a few studies were conducted for the discovery and comparison of the white root rot pathogen isolates in Malaysia. Examining virulence profiles of *R. microporus* isolates collected around Malaysia in contrasting disease reactions will contribute to a better understanding of the interactions underlying tolerance and susceptibility of rubber clone RRIM600 to different *R. microporus* isolates causing WRD. Determination of the most virulent isolate in present study is vital and shall be taken into consideration for the selection of suitable pathogen isolate in the development of more effective control measures in combating tenacious *R. microporus*. Taken together, field evaluation through visual observation and laboratory assays has led to screening of the most virulent isolate. It has been many years since the last published paper on the virulence profiles of *R. microporus* isolates in Malaysia has been reported. This study represents the most current report on the characteristics and virulence degree of *R. microporus* isolates causing WRD on rubber trees in Malaysia, by assessing the disease severity through the observation of the above- and below-ground symptoms.

The macroscopic characteristics of the white color and relatively fluffy aerial mycelia, with the milky color on the reverse side of the culture plate in the present study, have led to the preliminary identification of the five white root rot pathogen isolates as *R. microporus* which were also in agreement with previously reported description by Farhana et al. [6], Hood [19], Kaewchai and Soytong [1], and Nandris et al. [7]. Likewise, the microscopic results were similar to the findings by Kaewchai et al. [9] and Kaewchai and Soytong [1], who pointed out that the hypha of the fungal isolate of *R. microporus* were hyaline, septate, and possessed many branches without the presence of clamp connection. In contrast, the above observation was differentiable from the work done by Farhana et al. [6]. It was revealed that the *R. microporus* isolate was collected from rubber tree clone RRIM2020, containing a hyphae with clamp connection under compound microscope. In fact, clamp connections are unique structures to the fungi under the phylum Basidiomycota where *R. microporus* belongs to. Yet, the structure of clamp connection was not observed from any of the isolates in the present study. The function of clamp connections is to maintain the dikaryotic condition, and they were reported to form only in dikaryotic hyphae during their life cycle which was to ensure each of the cell is binucleate, but not all dikaryotic hyphae form them [20]. The morphological studies of *R. microporus* isolated from different rubber clones in Malaysia (RRIM2008, PB260, RRIM2024, PB350 RRIM600); the host from where the pathogen was isolated has provided information on the preliminary features of the white root rot pathogen.

The results of phylogenetic relationship in the present study demonstrated that the five isolates obtained from different rubber plantations in Malaysia were closely related to the isolates from Asian clade with 83% bootstrap support for their genetic similarity. Isolates from Asian clade were more closely related to the isolate from African clade but further away from those in South and Central America. It was clearly shown that the African and Asian clades were well distinguished from the clade consisting of isolates from South and Central America. Oghenekaro and partners [21] reported high levels of WRD occurrence in Asian and African rubber plantations where majority of the world’s rubber plantations are located such as Thailand, Indonesia, Sri Lanka, Nigeria, and Malaysia. On the contrary, this pathogen is less copious in native habitats of the rubber tree. According to Oghenekome [22], WRD is not a serious problem in rubber plantations in the South Americas, for example, Brazil, which was the native habitat of the rubber tree. Plantations represent even-aged monocultures and are more susceptible to disease than native forests, where epidemics are restricted by the age structure and the diversity of the plant community [23].

The RRIM600 cultivar in this study has clearly shown the symptoms of WRD infection just three months after inoculation, with foliar discoloration and root rotting incidences. These symptoms were similar with the previous works published by Kaewchai and Soytong [1] and Farid et al. [24], where the leaves turned yellow, and the infected roots turned into a darker color. The pathogenicity study revealed that RRIM600 rubber clone died in less than two weeks after the seedlings showed yellowing foliar symptom 3 months after inoculation. Omorusi [25] reported that the foliage symptoms usually appear only when the host is untreatable due to the fast and increased rate of disease infection. The discoloration of the leaves was usually linked to the disruption of the root function in the host. In root disease infection, wilting and defoliation of leaves are considered symptoms of advanced disease development [24].

It was interesting to note that those RRIM600 rubber seedlings infected with *R. microporus* isolate RL21, which is originally from Sarawak, had caused 83.33% of mortality on rubber seedlings in the sixth month after inoculation. As opposed to isolate RL21, the rest of the isolates have demonstrated a lower percentage of mortality rate on the rubber seedlings. These results explained that different isolates of the same species may cause different level of damage to the host, although the isolates are genetically closely related.

Despite being driven by the biotic factor (host and pathogen), Prasetyo et al. [26] stated that the development of the disease is also depends on abiotic factors such as humidity, temperature, pH, soil porosity, and soil characteristics. The cultural characteristics among *R. microporus* isolates proved to be quite variable in terms of their mycelial growth rates under different culture conditions. The effect of pH on the mycelial growth of *R. microporus* isolates in this study was conducted up to pH 7.0, as soil in Malaysia is usually acidic in nature [27]. The present in vitro study has revealed the significant effect of pH value on the growth rate of *R. microporus*. The highest mycelial growths for *R. microporus* isolates were recorded at pH 6.5 and the lowest mycelial growths were recorded at pH 3.5. Liyanage et al. [28] found that low pH value inhibited the growth of *R. microporus*. According to the findings by Rodesuchit et al. [29], Semangun [30], and Wahyuni et al. [31], the growth of *R. microporus* is favored by neutral pH (it can grow well in pH 6–7), whereas the fungal growth is slowed and suppressed at pH 4 and lower. Dede et al. [32] also reported that WRD occurrence are higher in areas with higher pH values than in areas with lower pH values, even within a rubber plantation. In the field, estate farmers have been amending the soil with sulfur to inhibit the growth of *R. microporus* with the means of reducing the use of pesticides or before the advent of fungicides. With very little detrimental impacts on the plant, the addition of sulfur can increase the acidity of the soil and is believed to help in slowing down the growth of *R. microporus* [33]. However, using pH to control the spread of *R. microporus* may not be an effective method, as detrimental effects of radial growth rate reduction were only observed in pH 3.5. Decent mycelial growth of *R. microporus* isolates were observed on media with pH higher than 3.5, especially isolate RL21 and RL22, which revealed their ability to tolerate wide range of pH.

Temperature is the most important environmental factor for regulating the growth and reproduction of fungi through its effect on conidial germination and appressorium development, and mycelial growth in this study. According to Oghenekaro et al. [34], *R. microporus* isolate (MS564b) collected from the sapwood of wild *Hevea brasiliensis* showed the highest mean growth on MEA at 25 °C, while the rest of the isolates (MUCL45064, ED310, and M13) have the maximum hyphae growth at 30 °C. From this study, it was clear that the growth of *R. microporus* isolates were harshly affected with temperature lower than 25 °C and above 30 °C. Mycelial growth for all isolates were inhibited when the temperature exceed 40 °C.

Liyanage et al. [28] reported that all the eleven isolates of *R. microporus* isolated in Sri Lanka grows best in continuous darkness except one isolate which grew well under both light and dark condition. In fact, some rubber planters are still implementing a long-adopted labor-intensive procedure to prevent WRD. The tap and part of lateral roots are expose to sunlight for few days prior to be painted with protective fungicides and covered back with fresh soil. This conventional prevention technique aims to create the environmental conditions that do not favor the disease development by combination of direct light exposure and high temperature which help to reduce the WRD occurrence [25]. However, such treatment is only complementary and not a substitute for complete elimination of the source of infection. As we could see from this study, light or dark incubation condition has no effect on the radial growth rate of all the isolates in this study except RL22, which showed a slightly lower growth rate when exposed under continuous light incubation. This explains that light has a very low inhibitory effect on *R. microporus* isolates found in this study.

Identifying the most virulent isolate will help to facilitate the long-needed studies on resistance research and host-related interactions on how the fungus is able to facilitate the decay of rubber tree. *R. microporus* isolate RL21 was found to have the highest growth adaptability to a wide range of culture conditions and this may cause difficulties to control the levels of pathogen inoculum in soil under real field conditions. The high disease severity index on rubber seedlings by isolate RL21 also depicted the potential of this isolate to be more harmful to rubber trees. Further information on this pathogen in Malaysia rubber plantations will be necessary for the successful management of WRD caused by *R. microporus.* A common strategy for controlling WRD is to reduce inoculum in the soil. Therefore, an appropriate technique for measuring the amount of *R. microporus* in the soil is essential for studying the effectiveness of different strategies for reducing soil inoculum levels.

## 5. Conclusions

Although all the five isolates of *R. microporus* collected from rubber plantations in different states of Malaysia are more than 98% genetically related, they possessed different degrees of virulence in causing the damage to RRIM600 rubber seedlings. *R. microporus* isolate RL21, originally from Sarawak, had caused 83.33% of mortality on rubber seedling at 6 MAI. Based on a higher mycelial growth rate on the culture mediums from the in vitro study and the high disease severity index on rubber seedlings, it can be presumed that capacity of *R. microporus* isolate RL21 to increase soil inoculum levels would be a fitness trait, especially on rubber plantation in Malaysia. It is important to determine which *R. microporus* strain have the potential to be more harmful to rubber tree in order to design a successful disease management strategy. Results from this study provide an insight in facilitating the decision making for disease intervention measures to combat the white root rot disease in rubber tree. The system approach should be applied to identify key points of vulnerability in the nursery operation where steps can be taken to minimize *R. microporus* risks. Proper disease control tactics cannot be implemented unless the correct disease problems are identified. It is therefore very important that nursery staff and plantation workers are well trained to recognize and manage *R. microporus*, as well as other pathogen species in the rubber plantation industry. Further studies on the phenotypic characteristic and metabolic profiling should be carried out to support the findings of the virulence level among the isolates of *R. microporus*.

## Figures and Tables

**Table 1:** Source of white root rot pathogen.

Isolate ID	Host (Clone)	Location
RL20	RRIM2008	RRIMINIS Seri Iskandar, Perak.
RL21	PB260	Sarikei, Sarawak.
RL22	RRIM2024	RRIM, Sungai Buloh.
RL25	PB350	Kg. Tohor, Negeri Sembilan.
RL26	RRIM600	Field 7C, Kota Tinggi, Johor.

**Table 2:** Evaluation of disease severity scoring based on the observations of above- and below-ground symptoms.

Scheme	Above-Ground Symptom	Below-Ground Symptom
0	Healthy, green leaves	Not found in the root fungal infections
1	1–25% yellow of foliage	Fungal infections on the root are less than 1%
2	26–50% wilting	1–10% fungal infections on the root
3	51–75% defoliation	11–50% fungal infections on the root
4	76–100% death of plant	51–90% fungal infection on the root
5	-	Fungal infections on the root of more than 90%

(Source: Wattanasilakorn et al. [18]).

**Table 3:** Disease severity index of rubber seedling clone RRIM600 based on above- and below-ground symptoms six months after being challenged with five different isolates of *R. microporus*.

Isolate ID	Disease Severity Index *		Average DSI of Above- and Below-Ground Symptoms
	Foliar Discoloration	Root Rotting	
RL20	52.08	80.00	66.04
RL21	76.04	85.00	80.52
RL22	57.29	80.00	68.65
RL25	31.25	55.00	43.13
RL26	43.75	65.00	54.38

* Note: Values of 24 plants.

**Table 4:** Mortality rate of rubber seedling clone RRIM600 inoculated with five different isolates of *R. microporus*.

Isolate ID	Mortality Rate per Month (%) *						Mortality Rate 6 MAI (%) *
	1	2	3	4	5	6	
RL20	0.00	16.67	20.83	12.50	8.33	8.33	66.66
RL21	0.00	20.83	25.00	16.67	12.50	8.33	83.33
RL22	0.00	12.50	12.50	16.67	16.67	12.50	70.84
RL25	0.00	4.17	8.33	12.50	12.50	12.50	50.00
RL26	0.00	12.50	12.50	12.50	12.50	12.50	62.50

* Note: Values of 24 plants.

**Table 5:** Average colony diameters of all five isolates of *R. microporus* after 3 days of incubation under different pH.

pH Value	Colony Diameter (mm)				
	RL20	RL21	RL 22	RL 25	RL26
3.5	15.42 ^f^	19.80 ^f^	12.19 ^d^	10.42 ^e^	8.49 ^f^
4	22.93 ^e^	34.34 ^e^	29.56 ^c^	16.63 ^d^	18.21 ^e^
4.5	25.34 ^de^	37.33 ^de^	31.16 ^c^	18.94 ^cd^	21.81 ^d^
5	27.53 ^cd^	38.24 ^cd^	31.41 ^c^	19.16 ^cd^	24.24 ^cd^
5.5	27.25 ^cd^	38.79 ^cd^	32.11 ^c^	21.66 ^c^	25.77 ^bc^
6	31.87 ^b^	43.05 ^b^	44.40 ^b^	36.89 ^a^	39.80 ^a^
6.5	38.32 ^a^	55.57 ^a^	52.65 ^a^	36.94 ^a^	39.96 ^a^
7	29.55 ^bc^	41.16 ^bc^	43.73 ^b^	29.48 ^b^	27.57 ^b^
*p*-value	<0.01	<0.01	<0.01	<0.01	<0.01

Note: means followed by the same letter in the same column are not significantly different at *p* ≤ 0.05 according to Tukey’s HRD test.

**Table 6:** Average colony diameters of all five isolates of *R. microporus* after 3 days of incubation under different temperature.

Temperature (°C)	Colony Diameter (mm)				
	RL20	RL21	RL 22	RL 25	RL26
20	18.51 ^c^	30.36 ^c^	21.34 ^c^	15.49 ^c^	11.93 ^c^
25	26.47 ^b^	35.57 ^b^	30.49 ^b^	20.65 ^b^	24.90 ^b^
30	30.45 ^a^	41.64 ^a^	35.62 ^a^	24.97 ^a^	27.11 ^a^
35	13.54 ^d^	21.07 ^d^	16.69 ^d^	13.62 ^c^	10.33 ^c^
40	0.00 ^e^	0.00 ^e^	0.00 ^e^	0.00 ^d^	0.00 ^d^
*p*-value	<0.01	<0.01	<0.01	<0.01	<0.01

Note: means followed by the same letter in the same column are not significantly different at *p* ≤ 0.05 according to Tukey’s HRD test.

**Table 7:** Average colony diameters of all five isolates of *R. microporus* after 3 days of incubation under different photoperiods.

Photoperiods	Colony Diameter (mm)				
	RL20	RL21	RL 22	RL 25	RL26
12 h Light and 12 h Darkness	27.34 ^a^	38.12 ^a^	31.54 ^a^	22.3 ^a^	27.53 ^a^
24 h Darkness	27.25 ^a^	38.79 ^a^	32.11 ^a^	21.66 ^a^	26.37 ^a^
24 h Light	25.19 ^a^	38.08 ^a^	27.85 ^b^	19.19 ^a^	26.87 ^a^
*p*-value	0.337	0.806	<0.01	1.125	0.738

Note: means followed by the same letter in the same column are not significantly different at *p* ≤ 0.05 according to Tukey’s HRD test.

## References

[1] Kaewchai S., Soytong K. (2010). Application of biofungicides against *Rigidoporus microporus* causing white root disease of rubber trees. J. Agric. Technol..

[2] Prasetyo J., Aeny T.N. (2013). The Preventive Control of White Root Rot Disease in Small Holder Rubber Plantation Using Botanical, Biological and Chemical Agents. J. Hama Penyakit Tumbuh. Trop..

[3] Sail R.M., Ahmad M. Enhancing socio-economy of rubber smallholders through effective transfer of technology. Proceedings of the National Rubber Economic Conference (NREC).

[4] Chaiharn M., Sujada N., Pathom-Aree W., Lumyong S. (2019). Biological control of *Rigidoporus microporus* the cause of white root disease in rubber using PGPRs in vivo. Chiang Mai J. Sci..

[5] Chee K.H. (1990). Recent development in rubber disease management. Proceedings of the 3rd International Conference on Plant Protection in the Tropics.

[6] Fatin Farhana A.H.K., Shamsul Bahri A.R., Vu Thanh T.A., Zakaria L. (2017). Morphological features of *Rigidoporus microporus* isolated from infected malaysian rubber clones. Malays. J. Microsc..

[7] Nandris D., Nicole M., Geiger J.P. (1987). Root rot diseases of rubber trees. Plant Dis..

[8] Holiday P. (1980). Fungal diseases of tropical crops. Australas Plant Pathol..

[9] Kaewchai S., Wang H.K., Lin F., Hyde K.D., Soytong K. (2009). Genetic variation among isolates of *Rigidoporus microporus* causing white root disease of rubber trees in Southern Thailand revealed by ISSR markers and pathogenicity. Afr. J. Microbiol. Res..

[10] Lu Q., Wang Y., Li N., Ni D., Yang Y., Wang X. (2018). Differences in the characteristics and pathogenicity of *Colletotrichum camelliae* and *C. fructicola* isolated from the tea plant [ *Camellia sinensis* (L.) O. Kuntze]. Front. Microbiol..

[11] de Jesus Eufrade Junior H., Ohto J.M., da Silva L.L., Lara Palma H.A., Ballarin A.W. (2015). Potential of rubberwood (*Hevea brasiliensis*) for structural use after the period of latex extraction: A case study in Brazil. J. Wood Sci..

[12] Pethin D., Nakkanong K., Nualsri C. (2015). Performance and genetic assessment of rubber tree clones in Southern Thailand. Sci. Agric..

[13] Woraathasin N., Nakkanong K., Nualsri C. (2017). Cloning and expression analysis of HbPR-1b and HbPR-3 in Hevea brasiliensis during inoculation with *Rigidoporus microporus*. Pak. J. Biol. Sci..

[14] Kaewchai S., Lin F.C., Wang H.K., Soytong K., Soytong K. (2010). Characterization of *Rigidoporus microporus* isolated from rubber trees based on morphology and ITS sequencing. J. Agric. Technol..

[15] Go W.Z., H’ng P.S., Wong M.Y., Tan G.H., Luqman Chuah A., Salmiah U., Toczyłowska-Mamińska R., Soni O., Wong W.Z., Chin K.L. (2015). Occurrence and characterisation of mycoflora in soil of different health conditions associated with white root rot disease in Malaysia rubber plantation. J. Rubber Res..

[16] Lin Y.H., Chang J.Y., Liu E.T., Chao C.P., Huang J.W., Chang P.F.L. (2009). Development of a molecular marker for specific detection of *Fusarium oxysporum* f. sp.. cubense race 4. Eur. J. Plant Pathol..

[17] Kumar S., Stecher G., Tamura K. (2016). MEGA7: Molecular Evolutionary Genetics Analysis Version 7.0 for Bigger Datasets. Mol. Biol. Evol..

[18] Wattanasilakorn S., Wattanasilakorn S., Sdoodee S., Nualsri C., Chuenchit S. (2012). Screening of rubber (*Hevea brasiliensis* Muell. Arg.) rootstocks for the white root disease resistance. Int. J. Agric. Technol..

[19] Hood I.A. The mycology of the basidiomycetes. Proceedings of the Australian Centre for International Agricultural Research (ACIAR) Proceedings.

[20] Zabel R.A., Morrell J.J., Zabel R.A., Morrell J.J. (2020). The characteristics and classification of fungi and bacteria. Wood Microbiology.

[21] Oghenekaro A.O., Miettinen O., Omorusi V.I., Evueh G.A., Farid M.A., Gazis R., Asiegbu F.O. (2014). Molecular phylogeny of *Rigidoporus microporus* isolates associated with white rot disease of rubber trees (*Hevea brasiliensis*). Fungal Biol..

[22] Oghenekome U.O. (2004). Natural rubber, *Hevea brasiliensis* (Willd. ex A. Juss.) Müll. Arg., germplasm collection in the Amazon Basin, Brazil: A retrospective. Econ. Bot..

[23] Burgess T.I., Wingfield M.J., Sivasithamparam K., Dixon K.W., Barrett R.L. (2002). Impact of Fungal Pathogens in Natural Forest Ecosystems: A Focus on Eucalyptus. Microorganisms in Plant Conservation and Biodiversity.

[24] Farid A.M., Lee S.S., Maziah Z., Patahayah M. (2009). Pathogenicity of *Rigidoporus microporus* and *Phellinus noxius* against four major plantation tree species in Peninsular Malaysia. J. Trop. For. Sci..

[25] Omorusi V.I., Dhal N.K., Sahu S.C. (2012). Effects of white root rot disease on *Hevea brasiliensis* (Muell. Arg.)—Challenges and control approach. Plant Science.

[26] Prasetyo J., Aeny T.N., Suharjo R. (2009). The corelations between white rot (*Rigidoporus lignosus* L.) incidence and soil characters of rubber ecosystem in Penumangan Baru, Lampung. J. Hama Penyakit Tumbuh. Trop..

[27] Rodríguez J.Á.C., Hanafi M.M., Syed Omar S.R., Rafii Y.M. (2009). Chemical characteristics of representative high aluminium saturation soil as affected by addition of soil amendments in a closed incubation system. Malays. J. Soil. Sci..

[28] Liyanage G.W., Liyanage A.D.S., Peries O.S., Halangoda L. (1977). Studies on the variability and pathogenicity of Rigidoporus lignosus. J. Rubber Res. Inst. Sri. Lanka.

[29] Rodesuchit A., Suchatgul S., Klaewklong B., Damnoi S. (2012). Efficacy of fertilizers to control white root disease of rubber caused by *Rigidoporus microporus* at the early planting stages. Rubber Thai. J..

[30] Semangun H. (2000). Penyakit-Penyakit Tanaman Perkebunan di Indonesia.

[31] Wahyuni M., Simanjuntak J.H., Sitompul I.O. (2018). Efektivitas fungisida berbahan aktif heksakonazol terhadap penyakit jamur akar putih bibit tanaman karet (*Hevea brasiliensis*). Agrotekma J. Agroteknologi Ilmu Pertan..

[32] Dede A.P.O., Akpaja E.O., Galillee J.E. (2011). Effect of pH on the growth of the white root rot pathogen, *Rigidoporus lignosus* (Klotzsch) Imazeki, on selected para rubber sustaining soils in Nigeria. Afr. Sci..

[33] Hashim I., Azaldin M.Y. (1985). Interaction of sulphur with soil pH and root diseases of rubber. J. Rubber Res. Inst. Malays..

[34] Oghenekaro A.O., Daniel G., Asiegbu F.O. (2015). The saprotrophic wood-degrading abilities of Rigidoporus microporus. Silva Fenn..

